# Exploring central sensitization symptoms in children with ADHD and their parents: a case-control study

**DOI:** 10.1186/s12888-026-08296-3

**Published:** 2026-06-19

**Authors:** Btissame Zouini, Sara Lundqvist, Abdennour El Mzadi, Rajna Knez, Anne-Katrin Kantzer, Ana Maria Sánchez Pérez, Nóra Kerekes

**Affiliations:** 1 Higher Institute of Nursing Professions and Health Techniques, Tetouan, Morocco; 2 Department of Biology, Faculty of Sciences, Abdelmalek Essaadi University, Tetouan, Morocco; 3 Centre for Holistic Psychiatry Research (CHoPy), Mölndal, Sweden; 4 Department of Pediatric Neurology and Psychiatry, Sahlgrenska University Hospital, Region Västra Götaland, Gothenburg, Sweden; 5 Institutions of Neuroscience and Physiology, University of Gothenburg, Gothenburg, Sweden; 6 Child and Adolescent Psychiatry, Skaraborg Hospital, Skövde, Sweden; 7 School of Health Science, University of Skövde, Skövde, Sweden; 8 Child- and Adolescent Psychiatry, NU Hospital Group, Trollhättan, Sweden; 9 Department of Medicine, University Jaume I, Castellon, Spain; 10 Institution of Health Sciences, University West, Trollhättan, Sweden

**Keywords:** Attention deficit hyperactivity disorder (ADHD), Central sensitization (CS), Children, Holistic care, Pain, Parent–child dyad

## Abstract

**Background:**

Emerging research suggests a link between attention deficit hyperactivity disorder (ADHD) and central sensitization (CS), a condition characterized by heightened sensitivity to pain and sensory stimuli. This study aimed to measure the severity of CS symptoms in children with ADHD and in their parents; compare results with those of a neurotypical group; and explore associations between CS symptoms and pain intensity as well as potential familial patterns.

**Methods:**

Participants included 37 children with ADHD (mean age = 11.54 years; 62.2% male) and 29 neurotypical children (mean age = 13.07 years; 58.6% male). Children completed the Central Sensitization Inventory (CSI) – Child and Adolescent Version and rated their pain intensity at the time of assessment and over the past week. One parent per child completed the adult version of the CSI.

**Results:**

Children with ADHD reported significantly higher CSI-indexed CS symptom scores than those in the comparison group (*p* < 0.001). In categorical analyses, neurotypical children were significantly more likely to fall into the subclinical (lowest symptom) CS range (adjusted *p* < 0.005). Children with ADHD also reported significantly greater pain intensity both currently (*p* = 0.016) and over the past week (*p* = 0.003). Stronger correlations between CSI-indexed CS and self-reported pain were observed in the ADHD group, particularly for current pain (ρ = 0.66, *p* = 0.002). Parents of children with ADHD also had elevated CS scores (*p* = 0.003), with the majority falling in the severe range. A moderate correlation between parent and child CSI-indexed CS scores was observed across the entire sample (ρ = 0.47, *p* = 0.002), while a strong correlation between parents’ CS scores and their own medical conditions was found only in the ADHD group (ρ = 0.66, *p* = 0.002).

**Conclusions:**

Children with ADHD, and their parents, reported elevated CSI-indexed CS symptoms and pain intensity, suggesting that CS symptoms may represent a clinically relevant feature in some ADHD presentations. These findings support the value of considering sensory and pain-related assessments in ADHD evaluations and highlight the need for further research into potential mechanisms and integrative interventions that may improve outcomes and reduce functional impairment in pediatric neuropsychiatric conditions.

**Clinical trial number:**

Not applicable.

## Background

The concept of central sensitization (CS) was first proposed by Clifford Woolf in 1983 [1], who described how neuroplastic changes in the central nervous system (CNS) can lead to an intensified pain response. According to the revised definition of pain by the International Association for the Study of Pain (IASP), pain is now defined as “an unpleasant sensory and emotional experience associated with, or resembling that associated with, actual or potential tissue damage” [2]. Although pain is a subjective sensory and emotional experience, CS refers specifically to the amplification of neural signaling within the CNS, which can lead to heightened pain sensitivity, even in the absence of ongoing tissue damage. Research on CS has since been well established through both animal models and human studies. Animal studies have uncovered the neurobiological mechanisms behind this phenomenon, including glial cell activation [3, 4], neurotransmitter imbalances, and the release of proinflammatory cytokines [5, 6]. Human research has demonstrated the role of CS in chronic pain conditions in adults, such as fibromyalgia, migraines, and irritable bowel syndrome, showing how altered pain processing in the brain and spinal cord leads to hypersensitivity [7–11].

Although the concept of CS has been well established in adult pain research [1, 6], it remains relatively underexplored in pediatric populations. Emerging evidence suggests that children and adolescents with chronic pain or neurodevelopmental conditions may exhibit heightened pain sensitivity, altered nociceptive processing, and reduced pain inhibition, which may reflect CS-related mechanisms [12–14]. Children with attention-deficit/hyperactivity disorder (ADHD) frequently exhibit sensory dysregulation, suggesting a potential link to CS. They may experience hypersensitivity to sound, touch, or light [15]; heightened pain perception [16]; and difficulty filtering sensory input [17, 18]. This heightened sensory response aligns with the mechanisms of CS, in which the central nervous system becomes hyperresponsive to stimuli, amplifying sensory and pain signals [19]. As a result, normally tolerable sensations may become overwhelming, contributing to emotional dysregulation and behavioral challenges often observed in ADHD [17, 20, 21] .

While CS and sensory dysregulation share overlapping features, both involving increased responsiveness to sensory input, CS specifically refers to central amplification of sensory signaling within the central nervous system, independent of peripheral injury [5]. Clarifying how these processes manifest in children with ADHD may therefore enhance understanding of the neurophysiological and sensory mechanisms underlying pain perception and sensory sensitivity in this population. Due to the limited availability of validated assessment instruments for children in this field, we employed a child- and adolescent-adapted version of the Central Sensitization Inventory (CSI) to address our research question [22]. Although elevated CSI scores are indicative of a CS phenotype, they do not constitute a definitive diagnosis of CS. Accordingly, throughout the present analysis, we use the term *CSI-indexed CS symptoms* to clarify that the CSI reflects a symptom phenotype associated with CS rather than providing a direct measure of neurophysiological sensitization.

Although ADHD and autism spectrum disorder (ASD) frequently co-occur, emerging evidence suggests that CS may be specifically linked to ADHD rather than ASD [23, 24]. Given that ASD is one of the most frequently coexisting neurodevelopmental disorders in ADHD—with potential overlap in sensory and pain-related features [25]—it was considered clinically relevant to examine its role in relation to CS. Research in adults has indicated that sensory processing abnormalities are key features of ADHD, particularly in females, regardless of co-occurring autistic traits [26]. This distinction is further supported by findings in adolescents, as the presence of ASD did not amplify pain prevalence in individuals with ADHD [27]. Notably, studies focusing on ADHD and chronic pain have identified a direct association with CS. For example, Ibrahim and Hefny [23] found that adults with ADHD and chronic back pain exhibited heightened CS, suggesting that individuals with ADHD may experience increased CS in the context of chronic pain. However, whether CS plays a mediating role between ADHD and altered pain perception remains to be clarified in studies including appropriate comparison groups. These findings suggest that individuals with ADHD, even without comorbid ASD, may be predisposed to CNS hyperexcitability, leading to increased pain sensitivity and sensory dysregulation. Critically, this points to a unique ADHD-CS pathway, distinct from autism-related sensory profiles, which may drive susceptibility to chronic pain conditions [23].

Sensory over-responsivity may be linked to co-occurring anxiety in ADHD, possibly due to shared mechanisms of central hyperexcitability and impaired regulation within limbic-prefrontal circuits [27, 28]. Additionally, some studies have observed that ADHD medication -particularly stimulant medications such as methylphenidate and amphetamines - may influence pain perception in patients with ADHD [29, 30], as it could target both the core symptoms of ADHD and CS [31]. However, other studies have reported no association between ADHD medication and pain frequency or intensity in adolescents [27]. These findings highlight the complex interplay between sensory processing, chronic pain, and ADHD symptomatology, which suggests that further investigation into sensory modulation alterations in ADHD populations is needed.

Of importance, research has suggested that parents of children with ADHD often experience chronic pain conditions, indicating a potential familial link between ADHD and CS [32, 33]. These epidemiological and clinical observations form the basis for exploring the relationship between CS in parents and their offspring with ADHD. Furthermore, parental ADHD itself has been identified as a salient biopsychosocial risk factor for the development of ADHD in offspring. A systematic review and meta-analysis by Uchida et al. (2020) [34] reported that children of parents with ADHD have a markedly higher risk of developing the disorder, supporting strong familial and potentially heritable mechanisms. Exploring the co-occurrence of ADHD and CS may therefore help identify shared neurobiological pathways, such as altered dopaminergic modulation, stress reactivity, and central hyperexcitability, that could underlie both attentional and sensory dysregulation. Familial clustering of ADHD, chronic pain, and CS-related traits supports the hypothesis of a shared inherited vulnerability that spans both emotional and sensory domains.

Systematic reviews have estimated that the global prevalence of ADHD in children and adolescents ranges between 5% and 8% [35, 36]. Given the high prevalence of ADHD and the likelihood that many of these individuals also have CS symptoms, it seems reasonable to assume that CS has a common impact on daily functioning in child psychiatric patients. Based on previous research and the study aims, we hypothesized that (1) children with ADHD would report higher CSI indexed CS symptom severity than neurotypical comparison children, and (2) higher CSI indexed CS scores would be associated with greater self-reported pain intensity. Regarding parents, (3) parents of children with ADHD are expected to show higher CSI indexed CS symptoms than parents of neurotypical children, and (4) parental CSI indexed CS scores would correlate positively with both the number of CS-related diagnoses and their child’s CS scores, indicating potential shared patterns within families.

## Methods

### Study design

This study is derived from a larger cross-sectional study examining the associations between ADHD, pain, inflammatory markers, and quality of life in children and adolescents, the design and methods of which are described in the previously published study protocol [24]. Given the breadth of the overall project, its findings are reported across separate publications, each addressing a distinct set of outcomes. The present paper focuses specifically on CS and pain-related outcomes.

CS was investigated among children and adolescents with and without ADHD, as well as their parents. The data were collected using standardized surveys distributed to participants at home. The study included two distinct participant groups: a clinical ADHD group and a nonclinical comparison group.

The clinical group was recruited from two child and adolescent psychiatric outpatient clinics in the Västra Götaland region of Sweden. Data collection for this group took place between November 2023 and June 2024. The comparison group was recruited from the general population during the same period. Because of a low response rate from September 2024 to February 2025, recruitment for the comparison group was redirected toward parents employed or studying at higher education institutions or working within the healthcare system, using paper announcements to distribute study information. Families expressing interest and meeting eligibility criteria (described in the next section) received survey materials by post.

The survey packets were mailed to participating families and contained separate child and parent questionnaires, along with a prepaid return envelope. Participants provided informed consent prior to participation. Whereas the clinical group’s data were pseudonymized to allow linkage with diagnostic and clinical information, the comparison group’s data were collected anonymously.

The final sample size for this study was determined pragmatically, based on feasible data collection over a two-year period. While the achieved sample was smaller than planned and insufficient for the originally planned subgroup analyses (e.g., stratification by sex, age, and comorbid ASD status), it was sufficient to detect several statistically significant differences and associations, particularly those with medium to large effect sizes.

### Study population

#### Clinical group

Children and adolescents were eligible for inclusion in the clinical group if they were between 7 and 18 years of age, had been diagnosed with ADHD within the past six months, and demonstrated adequate proficiency in Swedish. The diagnostic assessments were conducted in accordance with the Swedish National Board of Health and Welfare’s national guidelines for ADHD and autism [37]. Exclusion criteria for the clinical group included any diagnosis of intellectual disability, psychotic disorders, bipolar disorder, anorexia nervosa, bulimia nervosa, or substance use disorder as well as current suicidal ideation or behavior. Families in the clinical group received oral and written information about the study during their regular visits to the participating clinics. Participation was voluntary, and informed consent was obtained from both parents and children.

Data from 38 children with ADHD and their parents were collected; however, one family reported serious physical health conditions that could influence CS outcomes and pain perception, so it was excluded from the present analyses. The final clinical group consisted of 37 children (62.2% male; 37.8% female; mean age = 11.54 years, SD = 2.73), of whom 13 (35.1%) had coexisting ASD and one (2.7%) had coexisting anxiety. One parent of each participating child also completed the parent survey. Parents’ sociodemographic data were not collected.

#### Comparison group

The comparison group consisted of children and adolescents aged 7 to 18 years who had not been diagnosed with ADHD or any other serious psychiatric condition. To be included, both the child and the parent were required to have adequate proficiency in Swedish. Exclusion criteria were identical to those applied to the clinical group and included any diagnosis of ADHD.

The comparison group consisted of 32 children. While no participants in the comparison group reported psychiatric diagnoses, three children presented with serious physical health conditions. To minimize the potential confounding effects of chronic physical illness on CS scores, these participants were excluded from the present analyses. The final comparison group consisted of 29 children (58.6% male; mean age of 13.07 years, SD = 3.06), whose sex distribution did not differ significantly from that of the ADHD group (*p* = 0.77). However, the comparison group was significantly older than the clinical group (*p* = 0.049). One parent of each participating child also completed the parent survey. Parents’ sociodemographic data were not collected.

Participant recruitment was voluntary and based on convenience sampling, with no formal matching procedure applied. The final sample size was thus determined by the number of individuals providing consent.

### Measures

#### Central Sensitization Inventory - completed by parents

The Central Sensitization Inventory (CSI) is a two-part, self-report questionnaire designed to assess symptoms associated with CS in adults [38]. In the present study, the CSI was completed by parents to evaluate their own CS-related symptoms. Part A includes 25 items that measure physical and emotional symptoms, such as sensitivity to light, fatigue, and unrefreshing sleep. Each item is rated on a 5-point Likert scale ranging from 0 (“never”) to 4 (“always”), resulting in a total score between 0 and 100. Part B consists of a checklist in which participants indicate whether they have received a diagnosis of medical conditions commonly linked to CS, such as fibromyalgia or irritable bowel syndrome. The severity of CS symptoms, based on Part A scores, was interpreted using standard cut-off values: 0–29 (subclinical), 30–39 (mild), 40–49 (moderate), 50–59 (severe), and 60–100 (extreme). The Swedish translation of the CSI has previously been validated [39].

#### Central Sensitization Inventory – Child and Adolescent Version

The CSI Child and Adolescent Version (CSI-CA) is a child-friendly adaptation of the CSI designed for self-assessment of CS symptoms in children and adolescents aged 7 to 18 years [22]. It contains only the Part A section of the adult version, with modifications to language and formatting to ensure accessibility for younger respondents. Due to a technical error during survey distribution in the present study, the final item was omitted, resulting in a 24-item version of CSI-CA in our study with a maximum possible score of 96. To preserve interpretability, the severity categories were proportionally adjusted: 0–27 (subclinical), 28–37 (mild), 38–47 (moderate), 48–56 (severe), and 57–96 (extreme). A Swedish translation of the CSI-CA was used in this study. Evidence for adolescent use of the CSI is primarily derived from chronic musculoskeletal pain samples; applicability to neurodevelopmental cohorts such as ADHD remains to be established. Although this version has not yet undergone formal psychometric validation, it was linguistically adapted from the adult version and reviewed by a team including child psychiatrists and clinical personnel. The review process focused on ensuring age-appropriate language, clarity of symptom descriptions, and cultural relevance. Minor wording adjustments were made to enhance comprehensibility for children aged 7–18. No changes were made to the core symptom content of the items.

#### Pain intensity

Pain intensity was assessed using a scale ranging from 0 to 10, where 0 indicated “no pain at all” and 10 represented “unbearable pain.” The children were asked to rate the current intensity of their primary pain as well as their average pain intensity over the past week.

### Data analysis

All statistical analyses were performed using SPSS Statistics version 26.0 (IBM, Armonk, NY, USA). Statistical significance was set at *p* < 0.05, and all tests were two-tailed. Nonparametric tests were applied, because the Shapiro-Wilk test indicated that the assumption of normality was not met.

### Analyses corresponding to hypotheses about children

Group differences in CSI-indexed CS symptom scores and in pain intensity between the ADHD and comparison groups were assessed using the Mann-Whitney U test.

Correlations between child CSI-indexed CS scores and pain intensity (current and past-week ratings) were examined using Spearman’s rank-order correlation coefficient (ρ).

### Analyses corresponding to hypotheses about parents

Differences in parent-reported CS indexed CS symptom scores between parents of children with ADHD (PAC) and parents of neurotypical children (PNC) groups were also analyzed using the Mann–Whitney U test.

Associations between parent-reported CS indexed CS scores, their own CS-related diagnoses (Part B), and their child’s CS indexed CS scores were examined using Spearman correlations.

To control for the accumulation of alpha error resulting from multiple Chi-square tests across the five CSI severity categories, p-values were adjusted using the Holm–Bonferroni procedure (*α* = 0.05). Adjusted *p*-values are reported.

The strength of the correlations was interpreted according to standard conventions [40]. Correlation coefficients with absolute values between 0.00 and 0.19 were considered very weak, between 0.20 and 0.39 were considered weak, between 0.40 and 0.59 were considered moderate, between 0.60 and 0.79 were considered strong, and between 0.80 and 1.00 were considered very strong.

## Results

### Central sensitization symptom severity in children with and without ADHD

Sixteen children in the ADHD-only group and two in the comparison group did not fully complete the CSI, resulting in attrition rates of 43% and 7%, respectively. Children with ADHD-only diagnosis reported higher levels of CSI-indexed CS scores (*M* = 42.08, *SD* = 18.07) compared to those with ADHD and coexisting ASD (*M* = 37.13, *SD* = 13.03). However, this difference did not reach statistical significance (*p* = 0.84). Therefore, no attempt was made to form a subgroup of children with ADHD and coexisting ASD in subsequent analyses.

Children with ADHD reported markedly higher CSI-indexed CS mean scores than the comparison group, with mean scores exceeding double those of the comparison group (Table 1). This difference was statistically significant (*p* < 0.001), suggesting greater prevalence of heightened sensitivity to physical and emotional stimuli among children with ADHD.

**Table 1:** Children reported CSI-indexed CS symptom scores and severity ranges according to groups

Central sensitization		**Comparison** **(n = 27)**	**ADHD** **(n = 21)**	U	Z	*p*-value	
Total score	Mean	15.59	40.19	54.5	−4.76	**< 0.001**	
				**Chi-square test**			
				χ^2^	Cramer’s V	*p*-value	Adjusted *p*-value (Holm)
Severity ranges	Subclinical CS symptoms (%)	85.2	28.6	15.83	0.57	**< 0.001**	**< 0.005**
	Mild CS symptoms (%)	11.1	23.8	1.37	0.17	0.24	0.48
	Moderate CS symptoms (%)	3.7	9.5	0.68	0.12	0.41	0.48
	Severe CS symptoms (%)	0	19	5.61	0.34	**0.02**	0.08
	Extreme CS symptoms (%)	0	19	5.61	0.34	**0.02**	0.08

CS = central sensitization symptoms assessed by CSI-CA inventory

comparison = children without ADHD; ADHD = children with ADHD diagnosis

Categorizing children’s CS symptom scores by severity levels revealed that a greater proportion of children with ADHD fell in the severe to extreme range, while children without ADHD were more likely to be classified as subclinical. This distribution demonstrated a relatively strong effect size (Cramer’s V = 0.57; Table 1). Following the Holm–Bonferroni correction for multiple comparisons, only the subclinical category difference remained statistically significant (adjusted *p* < 0.005; Table 1), with children without ADHD being significantly more likely to report subclinical symptom levels – indicating lower overall CSI-indexed CS symptom burden in this group.

### Association between central sensitization and pain intensity in children with and without ADHD

**Fig. 1:**
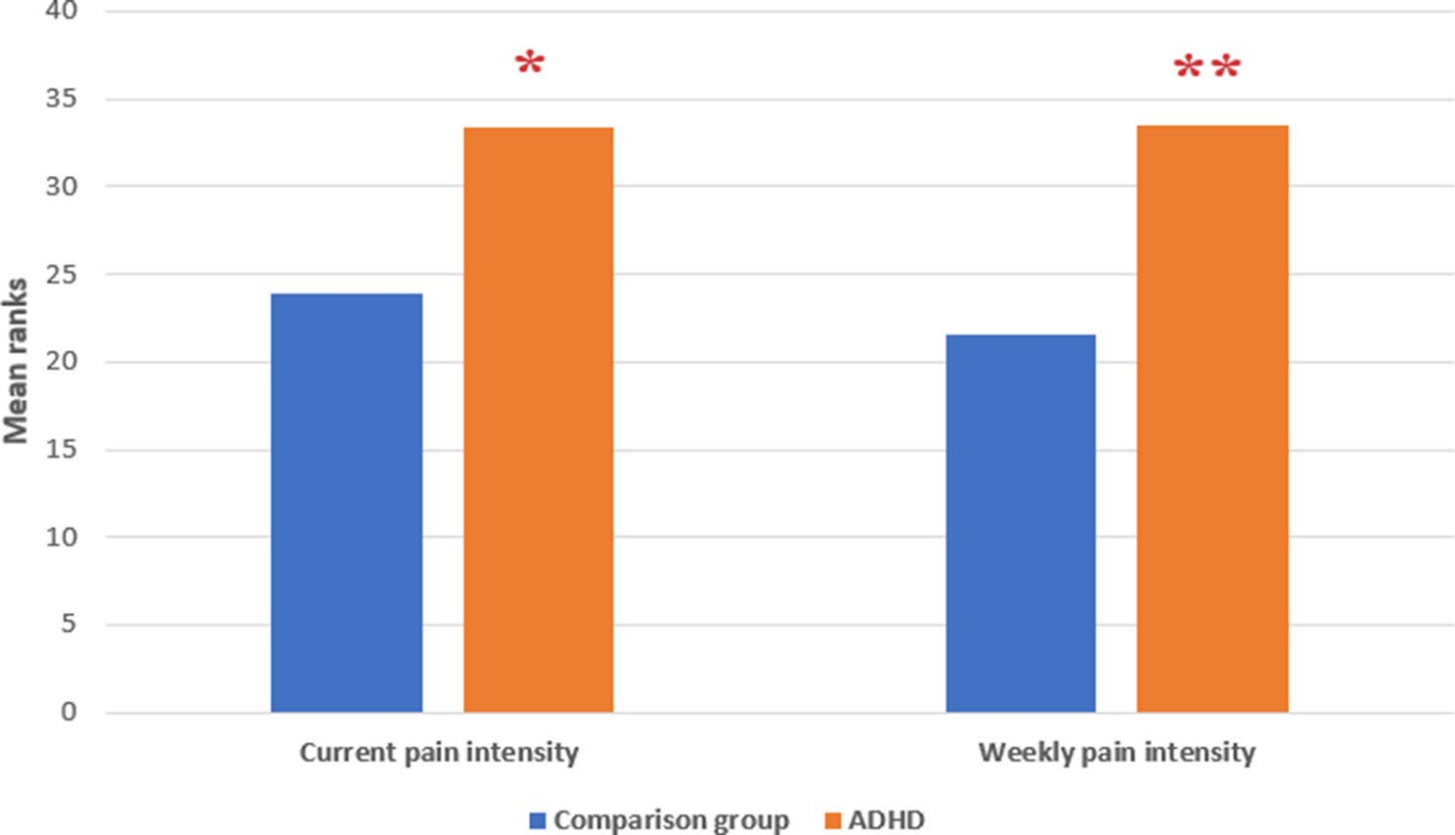
Significant differences in mean ranks of current and weekly pain intensity in ADHD vs. comparison groups. Values are presented as mean ranks from the Mann–Whitney U test. * *p* < 0.05 ** *p* < 0.01

There was a significant difference in current pain intensity between children in the ADHD group and the comparison group (Fig. 1; *p* = 0.016). Children with ADHD (*n* = 27) reported higher pain intensity at the time of assessment (*M* = 2.93, *SD* = 2.39, Median (*Md)* = 2) compared to the comparison group (*n* = 29; *M* = 1.76, *SD* = 1.57, *Md* = 1). A similar significant difference was observed for pain experienced during the past week (Fig. 1; *p* = 0.003). The ADHD group (*n* = 27) reported higher weekly pain intensity (*M* = 3.93, *SD* = 2.53, *Md* = 3) than the comparison group (*n* = 27; *M* = 2.15, *SD* = 2.09, *Md* = 1). Regarding pain intensity at the time of reporting, a strong correlation with CSI-indexed CS scores was found in the ADHD group (*ρ* = 0.61, *p* = 0.007), while a moderate correlation was observed in the comparison group (*ρ* = 0.52, *p* = 0.006). The association between pain intensity during the past week and CS scores was moderate and statistically significant in both the ADHD (*ρ* = 0.49, *p* = 0.039) and comparison (*ρ* = 0.45, *p* = 0.025) groups.

### Central sensitization symptoms in parents of children with and without ADHD

Eighteen parents from the ADHD group and one from the comparison group did not fully complete the CSI, resulting in attrition rates of 49% and 3%, respectively.

Consistent with the findings among children, parents of children diagnosed with ADHD (PAC group) reported significantly higher levels of CSI-indexed CS scores than parents of children without ADHD (PNC group) (Mann–Whitney U test, *p* = 0.003), indicating a substantial elevation in symptom burden among parents of children with ADHD (Table 2).

**Table 2:** Parent-reported CS and severity ranges of CS according to groups

				Mann–Whitney U test			
Central sensitization		PNC (28)	PAC (19)	U	Z	*p*-value	
Total score	Mean	17.93	31.74	131	-2.93	**0.003**	
				**Chi-squared test**			
**Central sensitization**		PNC	PAC	χ^2^	Cramer’s V	*p*-value	Adjusted p-value (Holm)
Severity ranges	Subclinical CS symptoms (%)	72.4	52.6	2.52	0.23	0.11	0.44
	Mild CS symptoms (%)	6.9	15.8	0.89	0.14	0.35	0.6
	Moderate CS symptoms (%)	17.2	5.3	1.61	0.19	0.20	0.6
	Severe CS symptoms (%)	0	21.3	6.44	0.37	**0.010**	0.05
	Extreme CS symptoms (%)	0	5.3	1.51	0.18	0.22	0.6

CS symptoms measured by CSI-A inventory

PNC = parents of neurotypical children

PAC = parents of children diagnosed with ADHD

Categorizing parents’ CSI-indexed CS symptom scores by severity level (where the subclinical category represents the lowest level of CS symptoms, indicating minimal symptom burden) revealed a significant association between group membership and CS symptom severity, with a moderate effect size (*p* = 0.01; Table 2). This association did not, however, remain statistically significant after applying the Holm–Bonferroni correction (*adjusted p* = 0.005).

### Parent–child associations of CS symptom scores and parental diagnoses

Parent-reported CS symptom scores were significantly associated with both parents’ own CS-related health status (CSI-B) and their children’s CS symptom scores (Table 3).

**Table 3:** Correlations between parent- and child-reported central sensitization (CS) symptom scores and parental CS-related diagnoses (CSI-B)

Group	Correlation	ρ	*p*-value
Full sample (ADHD + comparison)	Parent CS × Child CS	0.47	**0.002**
Parents of children with ADHD	Parent CS × Number of diagnoses (CSI-B)	0.66	**0.002**
Parents of children without ADHD	Parent CS × Number of diagnoses (CSI-B)	−0.10	0.62

ρ = Spearman’s rank-order correlation coefficient

Across the full study sample (both groups), there was a moderate, positive correlation between parents’ and their children’s CS scores (*ρ* = 0.47, *p* = 0.002), suggesting a shared pattern of symptom severity within parent–child dyads. That is, higher CSI-indexed CS symptom scores in parents tended to be associated with higher CSI-indexed scores in their children, indicating that symptom levels moved in the same direction. In addition, among parents of children with ADHD, the association between the number of their diagnosed diseases (assessed by the CSI-B) and their CSI-indexed CS scores was strong and statistically significant (*ρ* = 0.66, *p* = 0.002). However, this relationship was not statistically significant among parents in the comparison group (*ρ* = −0.10, *p* = 0.62).

## Discussion

This study provides preliminary evidence of elevated self-reported CSI-indexed central sensitization (CS) symptoms among children diagnosed with ADHD and their parents. Children in the ADHD group reported higher CSI-indexed CS symptom scores than peers without ADHD, suggesting a greater overall burden of CS-related symptoms in this population. Although a higher proportion of children with ADHD fell within the severe to extreme symptom ranges, only the difference in the subclinical category - reflecting the lowest symptom levels - remained statistically significant after Holm–Bonferroni correction for multiple comparisons. This finding indicates that children without ADHD were more likely to report minimal CS symptoms, while differences across higher severity categories followed similar trends but did not withstand correction for multiple comparisons. Notably, co-occurring ASD did not appear to further influence CS levels, as children with both ADHD and ASD reported CS symptoms comparable to those with ADHD alone. These findings suggest that, within this cohort, CS symptoms may not be specific to a single diagnostic category and could represent a transdiagnostic feature across neurodevelopmental disorders [41]; though this interpretation requires confirmation in larger, diagnostically diverse samples. These findings align with previous literature on sensory processing difficulties and heightened pain sensitivity in ADHD, independent of co-occurring ASD [24], and suggest that CS may constitute a relevant mechanism contributing to the clinical complexity of ADHD. While not exclusive to ADHD, it may be particularly pronounced or clinically salient in this population.

Notably, children with ADHD reported significantly higher pain intensity, both at the time of assessment and when reflecting on the past week. While these brief self-report measures offer a limited view of the pain experience, they are relevant in the context of CS, where heightened pain perception is a core feature. However, the current study did not aim to provide a comprehensive assessment of pain. Rather, these findings should be interpreted as preliminary and supportive of a broader clinical pattern.

The neurobiological underpinnings of these findings are supported by studies implicating shared brain regions in ADHD and chronic pain, where converging evidence from neuroimaging studies identifies consistent abnormalities in pain processing and attentional networks, particularly involving the anterior cingulate cortex, prefrontal cortex, thalamus, and insula [42, 43]. These regions demonstrate both structural alterations and functional dysregulation in ADHD populations, suggesting a shared neural substrate for attentional deficits and altered nociceptive processing. Of particular pathophysiological significance is the role of dopaminergic neurotransmission, which appears to mediate a dual influence on both cognitive function and pain perception through its modulatory effects on microglial activity and neuroinflammatory pathways [44–46].

Neuroinflammatory pathways have increasingly been recognized as relevant to ADHD and chronic pain. Microglial activation and elevated proinflammatory cytokines can contribute to central hyperexcitability, and rodent models indicate that targeting neuroinflammation can reduce both stimulus sensitivity and other ADHD symptoms [3, 4]. These mechanisms highlight promising avenues for integrative treatments, such as mindfulness practices or physical exercise, that may simultaneously target inflammation, pain, and attentional regulation [44–46]. Further supporting the inflammation hypothesis underlying ADHD symptoms, recent evidence has shown that children with ADHD display blood metabolome alterations, pointing to connections between microbiota dysbiosis, oxidative stress, and the dopaminergic pathway [47].

Our findings further demonstrate that pain intensity and CSI-indexed CS symptom levels are more strongly correlated in the ADHD group, particularly for pain reported at the time of assessment. The strength of these associations suggests that CS symptoms may play a direct role in modulating pain perception in children with ADHD. These results underscore the possibility that shared neurobiological mechanisms—such as dopaminergic dysfunction and neuroinflammation—contribute not only to attentional difficulties [48–50] but also to increased pain sensitivity and sensory dysregulation in this population.

The familial patterns emerging from our data support a potential endophenotypic model of CS symptoms in ADHD. The moderate but significant parent–child correlations in CSI-indexed CS symptoms align with the established heritability of both ADHD (approximately 70%–80%) [32, 51, 52] and pain sensitivity [53–55]. This pattern may reflect shared neurobiological vulnerabilities, particularly involving dopaminergic and serotonergic systems implicated in both conditions; however, no conclusions regarding inheritance or causality can be drawn from the present study.

Beyond potential genetic transmission, shared environmental and psychosocial factors may play a role in shaping CS symptom expression within families. Families of children with ADHD often report higher levels of chronic stress, irregular daily routines, and strained parent–child interactions [56, 57]. These stressors are known to dysregulate the hypothalamic-pituitary-adrenal (HPA) axis and increase susceptibility to CNS hyperexcitability [58–60].

From a clinical perspective, these preliminary findings point to the potential value of broadening current approaches to ADHD assessment and management. The observed association between ADHD and CSI-indexed CS symptoms suggests that questions about pain and pain-related experiences should be incorporated into ADHD assessment. Such features could contribute to a more comprehensive understanding of the individual’s symptom profile and support patient-centered care. Although replication of the present findings is warranted and the mechanisms underlying these associations remain to be fully elucidated, emerging hypotheses—including the roles of stress regulation, neuroinflammation, and central nervous system sensitization—warrant further investigation.

Taken together, these findings raise the possibility that CS symptoms might be more than a comorbid feature in some individuals with ADHD – potentially representing an integral part of the condition within a subset of cases. However, this interpretation should remain tentative until supported by longitudinal and mechanistic research. Future studies are needed to clarify the role of CS in ADHD’s heterogeneous presentation, as CS may be relevant to a person-centered understanding of ADHD in the individual patient.

### Strengths and limitations

This study provides preliminary evidence for an association between ADHD and CS symptoms in children and adolescents - an area that could have implications for clinical assessment treatment strategies. One of its strengths lies in the inclusion of a comparison group, which enables between-group comparisons. The successful recruitment of 37 children with ADHD, along with one parent per child, represents a notable achievement given the practical and emotional demands often faced by families managing ADHD. Furthermore, the inclusion of parental CS symptom data provides important insights into potential familial and intergenerational patterns of sensitization.

Nevertheless, several limitations warrant consideration:

### Methodological aspects

The relatively small sample size, coupled with high attrition rates - especially pronounced in the ADHD group (43% among children and 49% among parents) - limited statistical power and constrained the generalizability of the findings. A major methodological concern in this study was the low completion rate of the CSI, likely related to the voluntary nature of participation and the length of the questionnaire, which may have introduced selection bias by favoring a subset of respondents.

The CSI was completed at home without researcher supervision, which may have contributed to noncompletion, particularly in families managing ADHD-related challenges. This format also lacked structured support, which may have reduced engagement, particularly in a population where attentional difficulties or task fatigue are common [61].

At the same time, it is possible that individuals experiencing more pronounced symptoms, particularly pain or somatic complaints, were more motivated to participate and complete the questionnaires, potentially overrepresenting a more clinically burdened subgroup. This remains speculative but warrants further investigation. Importantly, all participants completed the CSI under comparable, real-world conditions, enhancing the ecological validity of the findings.

A central limitation of this study concerns the concept and operationalization of CS. The instrument used (CSI) is a self-report questionnaire that does not directly measure neurophysiological sensitization processes in the central nervous system. Instead, it captures a cluster of symptoms commonly associated with functional somatic syndromes, including widespread pain, fatigue, sensory hypersensitivity, sleep disturbances, and cognitive difficulties [38]. Moreover, while Part B of the adult version includes a list of medically diagnosed functional somatic syndromes, the child and adolescent version relies solely on symptom-based reporting. As such, the CSI provides an indirect estimate of CS symptom burden, which may overlap with other clinical phenomena, including emotional distress or attentional difficulties, particularly in neurodevelopmental populations. CSI severity thresholds were developed in adults; in youths, they should be considered exploratory and used for descriptive stratification rather than diagnostic classification.

This underscores the need for complementary objective measures of CS in future research, especially when working with pediatric samples. The present study relied exclusively on self-report instruments, which capture subjective perceptions of symptoms rather than direct neurobiological indicators of central sensitization. This limits construct validity and introduces potential bias, particularly in populations characterized by attentional or executive functioning difficulties.

Moreover, detailed information regarding the type, duration, and location of pain was not collected, which limits the contextual interpretation of CS symptom scores in relation to specific pain characteristics. Sensory dysregulation was also not directly assessed, restricting the ability to determine how abnormalities in sensory processing may relate to central sensitization in children with ADHD. The absence of objective sensory testing (e.g., Quantitative Sensory Testing [QST] and Conditioned Pain Modulation [CPM]) restricts construct validation; future pediatric studies should incorporate physiological indices alongside self-reported measures to clarify this relationship.

### Sample characteristics and group comparability

The wide age range of participants represents an additional limitation. Age-related differences may influence both central sensitization and pain thresholds, yet these effects could not be explored in the present sample due to its limited size. Future research should examine age as a potential moderating factor in the relationship between ADHD and CS.

A further limitation concerns the lack of formal matching between the ADHD and comparison groups. Because participants were recruited on a voluntary basis, the groups were not matched on demographic variables such as age or sex. Although no significant differences were observed between the groups in these variables, the absence of a strict matching procedure may still limit the comparability and generalizability of the findings.

### Diagnostic scope and contextual factors

An important limitation concerns the absence of a comparison group consisting solely of children with ASD. Because some participants in the ADHD group had comorbid ASD, the results cannot determine whether CS is specific to ADHD or shared with ASD. Future research including separate ADHD-only and ASD-only groups is warranted to clarify this.

We also lacked information on medication use among participants with ADHD, which may have influenced reported pain perception. Furthermore, no sociodemographic data were collected from parents, including sex, educational background, and health status. This limits our ability to interpret parent–child dynamics, examine contextual moderating factors, or assess the influence of socioeconomic status. The comparison group primarily consisted of children from families where parents had higher education, and possibly higher socioeconomic status, which may have contributed to lower CS symptom scores in that group and introduced a confounding effect. Socioeconomic disparities are known to affect pain perception, access to care, and exposure to stressors, all of which may have influenced the results [62, 63].

### Study design

The study’s cross-sectional design limits our ability to draw causal inferences or map developmental trajectories. Future longitudinal studies are needed to clarify the temporal and potentially bidirectional relationships between neurodevelopmental vulnerability, chronic stress exposure, and pain sensitization.

## Conclusion

This study provides preliminary evidence suggesting that self-reported CS symptoms may be a relevant factor in the clinical presentation of some children with ADHD and their caregivers. The findings highlight the potential value of considering somatic symptoms, such as pain perception and sensory dysregulation within a broader, more integrative framework of ADHD assessment and care.

Further research, particularly longitudinal and mechanistic studies, is needed to determine whether and how CS symptoms develop over time in ADHD populations and whether targeted interventions addressing sensory and somatic dimensions may enhance outcomes. Such approaches may benefit not only children but also their families, particularly in cases where shared symptom patterns are present.

## Data Availability

Our dataset includes both anonymized and pseudonymized data, some of which remain sensitive. Consequently, we cannot make the full dataset publicly available. Deidentified or anonymized data may be made available upon reasonable request to the corresponding author, contingent on ethical approval and data protection considerations.

## Declarations

### Study preregistration statement

This study was conducted in accordance with the protocol published in Kerekes N, Lundqvist S, Schubert Hjalmarsson E, Torinsson Naluai Å, Kantzer AK, Knez R. The associations between ADHD, pain, inflammation, and quality of life in children and adolescents—a clinical study protocol. *PLOS One*. 2022;17(9):e027365 [24]. The analyses presented here represent a planned part of the broader study design.
